# Formulation and Evaluation of Nanocrystals of a Lipid Lowering Agent

**Published:** 2016

**Authors:** Dina Louis

**Affiliations:** *Pharmaceutics Department. Faculty of Pharmacy, Cairo University., Cairo, Egypt.*

**Keywords:** Atorvastatin, Bioavailability, Hhomogenization, Nanocrystals, Stabilizer

## Abstract

Atorvastatin calcium, the lipid lowering agent, is taken as a model drug characterized by poor water solubility and bioavailability. In this study an attempt was made for preparation of nanocrystals using high pressure homogenization. A number of stabilizers were included as well as polymers at different concentrations, and the formulations were homogenized for ten cycles at a pressure of 1000 bars. The obtained nano crystals were evaluated by determining their size, zeta potential, saturated solubility and dissolution rate. Results revealed that Formulation 3, containing (10: 1) drug to sodium lauryl sulphate ratio, possessed the highest saturated solubility and dissolution rate, and hence was analyzed by X-ray diffraction analysis, differential scanning calorimetry, Fourrier transform infrared spectroscopy and scanning electron microscopy. An *in-vivo* study was carried out on the successful formulation in comparison to drug powder using rats as experimental animals. A significant increase in the area under the concentration-time curve C_pmax_ and MRT for nanocrystals was observed in comparison to the untreated atorvastatin calcium.

## Introduction

Atorvastatin, as a synthetic lipid-lowering agent, is an inhibitor of 3-hydroxy-3-methyl-glutaryl-coenzyme A (HMG-CoA) reductase which catalyzes the conversion of HMG-Co A to mevalonate, an early rate-limiting step in cholesterol biosynthesis (1). It is insoluble in an aqueous solution of pH 4 and below, very slightly soluble in water and slightly soluble at pH 7.4 phosphate buffer, slightly soluble in ethanol and freely soluble in methanol (2). Atorvastatin is readily permeable at the physiological intestinal pH, yet its oral bioavailability following a 40 mg oral dose does not exceed 12% (3).

Thus, attempts were made to improve the solubility of atorvastatin so as to improve its oral bioavailability. These attempts included formulation of atorvastatin spherical crystals (4), liquisolid compacts (5), spray dried atorvastatin calcium (6), atorvastatin nanoemulsions (7) and nanocrystals of atorvastatin (8).

Nano-sizing refers to the reduction of the active pharmaceutical ingredient particle size down to the sub-micron range. It refers to a reduction in size down to 100-300 nm in the pharmaceutical field, and sometimes the definition extends to include particles having a size up to 1000 nm (9). This reduction in particle size leads to a significant increase in the dissolution rate of the API (Active pharmaceutical ingredient), which in turn can lead to substantial increases in bioavailability (10). There are two main approaches to preparing nanoparticles: ‘top down’ and ‘bottom up’ technologies (11, 12). The former method involves mechanical attrition to reduce the size of large crystalline particles through wet milling and high pressure homogenization (13). The latter method utilizes controlled precipitation or crystallization (12).

A very important issue in nanocrystal formulation is the choice of the stabilizers, which are required to prevent nanoparticle aggregation. Within this reduced size range, attractive forces between particles appear. These attractive forces arise due to dispersion or van der Waals forces. As particles come close, these attractive forces increase and particle aggregation tends to become irreversible (14). The added stabilizers act to prevent or minimize such aggregation through steric or electrostatic stabilization. Steric stabilization is achieved by adsorbing polymers onto the particle surface, whereas electrostatic stabilization is obtained by adsorbing charged molecules, which can be ionic surfactants or charged polymers, onto the particle surface. Pharmaceutical excipients that can be used as stabilizers include the cellulosics, such as hydroxypropyl cellulose (HPC) and hydroxypropylmethyl cellulose (HPMC), povidone (PVP K30), and pluronics (F68 and F127); as well as surfactant stabilizers which can be non-ionic, such as polysorbate (Tween 80), or anionic, such as sodium lauryl sulfate (SLS) and docusate sodium (DOSS) (15, 16).

Formulated nanocrystals can be characterized through a number of tests including particle size determination as well as surface charge measurement (zeta potential), particle crystallization and dissolution (13, 17).

In this study, we aim at improving the poor water solubility of atorvastatin calcium, and hence its bioavailability, through size reduction to the nano-size range. The high pressure homogenization technique has been employed using a variety of stabilizers at various concentrations.

## Experimental

*Materials*

Atorvastatin Calcium, was kindly provided by Amoun Pharmaceuticals Co., Egypt. Sodium lauryl sulphate (SLS), tween 80, potassium acid phosphate, disodium hydrogen phosphate, ethyl acetate and orthophosphoric acid were purchased from Al Nasr Pharmaceutical Co., Egypt. Hydroxpropyl methyl cellulose (HPMC), Hydroxpropyl cellulose (HPC) and methanol HPLC grade were obtained from, Sigma-Aldrich, USA.

*Methods*

*Preparation of nanocrystals*

Table 1 gives the composition (ratio by weight) of the prepared nanocrystal formulations, using the surfactants sodium lauryl sulphate (SLS) and Tween 80 (18), as well as the polymers HPMC and HPC (10). The formulations were prepared by mixing 200 mg drug (18, 19), and the weighed amounts of stabilizers. This was followed by suspending the mixtures in 10 milliliters of water.

**Table 1:** Composition of the prepared nanoparticle formulations

**Formulation #**	**Composition**
1	20: 1 Drug :SLS
2	15: 1 Drug: SLS
3	10:1 Drug: SLS
4	5:1 Drug: SLS
5	20:1 Drug: Tween 80
6	15:1 Drug: Tween 80
7	10:1 Drug: Tween 80
8	5: 1 Drug: Tween 80
9	1:1 HPMC : Drug
10	0.5: 1 HPMC: Drug
11	1:1 HPC: Drug
12	0.5: 1 HPC: Drug

SLS: sodium lauryl sulphate; HPMC: hydroxypropyl methyl cellulose; HPC: hydroxypropyl cellulose

These suspensions were sonicated (Elmasonic S 30 H, Germany) for half an hour to break any agglomerated powder. This pre-milling process was followed by exposure to high pressure homogenization (Standsted SPCH-10- Pressure Cell Homogeniser, UK) for 10 cycles at a pressure of 1000 bar. The resulting suspensions were lyophilized (Savant Novalyphe-NL500, USA) to obtain dry powder (20).

*Evaluation of prepared formulations*

*Determination of particle size and zeta potential of formulations*

Appropriate dilutions of formulations using deionized water were prepared and then these dilutions were examined for their size and zeta potential using a zetasizer (Malvern Zetasizer Nano ZS, UK). The values were compared to those of the drug and a statistical analysis was carried out using a One-Way ANOVA (Post Hoc test used is the LSD) at p < 0.05, using SPSS 16 program.

*Determination of the saturated solubility of formulations*

Saturated solubility of the prepared formulations, the drug as well as the lyophilized drug was determined in water. An excess of the dried powder formulations was suspended in a fixed volume of water and was shaken in a constant temperature water bath (Stuart SBS 40, UK) at 100 rpm at 37 ± 0.5 ^o^C for 48 h (till equilibrium solubility was attained). At the end of the period, the samples were ultra-centrifuged at 15000 rpm (Megafuge 1.0R, Heraeus, Germany) for 15 min to remove the excess solid, appropriately diluted, and the concentration of atorvastatin was determined by UV spectrophotometry at 240 nm (4). The concentration of the dissolved atorvastatin was calculated using the equation derived from the built calibration curve of the drug in distilled water. The formulation of the highest solubility was compared to that of a physical mixture of the same drug to stabilizer ratio. The experiment was repeated three times for each formulation. A statistical analysis was carried out using a One-Way ANOVA at p < 0.05 to compare the results.

*In-vitro dissolution studies*

A USP dissolution tester -Apparatus II (Vankel, VK 7500, USA) was used to determine the dissolution profile of the tested formulations. An amount of each formulation equivalent to 10 mg atorvastatin was accurately weighed. The dissolution medium used was 1000 mL distilled water, at 37 ^o^C ± 0.5^ o^C and the paddles were operated at 50 rpm (21). Five milliliter samples were withdrawn from the dissolution medium after 5, 10, 15, 30, 60, 90 and 120 min. The samples were compensated for by equal volumes of distilled water, and these samples were centrifuged and analyzed for atorvastatin spectrophotmetrically. Each formulation was repeated three times. The dissolution profile of the plain drug was also determined, as well as that of the physical mixtures of formulations which possessed the highest dissolution rates. The data of the release experiments obtained at 30 min were compared by determination of the dissolution efficiency (%D.E) and a statistical analysis was run using a One-Way ANOVA test (Post Hoc test: LSD).

The dissolution efficiency was calculated according to the following equation (22)

Dissolution efficiency (DE) = t 0ʃy.dt / y100 t * 100

*X-ray Diffraction Analysis, Differential scanning calorimeter (DSC) and Fourrier transform infrared spectroscopy (FTIR)*

The formulation that proved to possess the best results in the above investigations was tested for the crystalline properties and compared to atorvastatin calcium powder (XRD, X’pert pro, Pan Analytical, Netherland) over a range of 2θ from 5^o^ to 50^o^ with Ni-filtered Cu-Ka radiation. The scan speed was 3 degree. min^-1^.

Differential scanning calorimetric examinations of both the plain drug and the successful formulation were done. The samples were heated at a constant rate of 10^o^C/min, in an atmosphere of nitrogen over a temperature range of 20-250^o^C using DSC-50 (Shimadzu, Kyoto, Japan). 

Similarly, The IR spectra of the pure drug, the successful formulation and its physical mixture were recorded using Infrared spectrophotometer (Shimadzu IR-345‑U-04, Japan).

*Scanning electron microscopy*

The best formulation was examined for its surface properties and compared to atorvastatin calcium powder (Jeol-JSM 5200 Scanning Microscope, Japan).

*In-vivo Studies*

An *in-vivo* pharmacokinetic study, on experimental rats, to compare the formulation of choice and the drug was carried out. This investigation adhered to the Principles of Laboratory Animal Care. Two groups, each containing six female albino rats (0.18–0.22 kg), were used for the test. The rats were fasted overnight, then they were allowed to administer 0.5 mL aqueous dispersion of Atorvastatin drug and the most successful formulation of Atorvastatin nanoparticles-formulation (equivalent to 10 mg/mL Atorvastatin) using oral feeding tube. Blood samples of 0.2 mL were withdrawn through the tail vein of rats after 0.5, 1, 1.5, 2, 2.5, 4, 6 and 24 h of sample administration. The withdrawn samples were centrifuged at 5000 rpm for 20 min. The plasma was separated and stored at -20 °C until drug analysis was carried out using an HPLC analytical method of analysis (23).

*HPLC Analysis*

The samples were analyzed using reversed-phase high performance liquid chromatography. The instrument used was HPLC Knauer, Germany, equipped with two pumps, RI detector, UV detector. The conditions for analysis involved a flow rate of 0.7 mL/min; the column used was Kinetex 2.6u C18 100X 4.6 mm; the temperature was kept constant at 50^o^C; the UV detector was operated at a wavelength of 247 nm; the mobile phase was 0.05 M sodium phosphate buffer: methanol (33: 67 v/v), adjusted to a pH of 4 with orthophosphoric acid 6M (24).

The plasma samples were prepared by adding 1ml ethyl acetate to 0.1 ml plasma, mixed by vortex for 30 sec at 2500 rpm (Stuart, UK) to extract the drug, centrifuge for 5 min at 5000 rpm. The upper layer was then separated, and evaporated to dryness at 40 ^o^C using a constant temperature water bath. The residue was then dissolved in 5 mL of the mobile phase to which a fixed volume of the internal standard (diclofenac sodium) solution (0.4 mg/10 mL) was added. All the samples were filtered through a 0.11 millipore size membrane filter before injection into the column. 

A calibration curve was constructed in rat plasma by spiking rat plasma with increasing amounts of atorvastatin calcium solution (4 mg/10 mL) to get concentrations of 40-360 µg/mL to which a fixed volume of the internal standard was added. The drug was extracted from these standard solutions in the same manner referred to in the sample preparation.

The pharmacokinetic parameters were calculated using Equiv. Test PK-C software, and were statistically compared using an independent Student-t test at p < 0.05.

## Results and Discussion

The obtained nanocrystals were examined according to the previously named tests and the results are exhibited as follows:

*Particle size and zeta potential*

The obtained formulations showed the following particle size and zeta potential recorded in Table 2.

**Table 2:** Particle size and zeta potential values of prepared formulations compared to the drug.

**Formulation**	**Particle size (nm)**	**Zeta potential (mv)**
1	583.7(±11.3)	-24.8(±1.5)
2	574.2(±10.04)	-23.2(±0.141)
3	546.1(±11)	-25.7(±0.42)
4	593.1(±9.26)	-26.2(±0.9)
5	611(±6.36)	-7.52(±0.97)
6	539.2(±10.04)	-26.1(±0.97)
7	502.5(±2.47)	-25.4(±1.48)
8	231.7(±12.9)	-27.9(±1.6)
9	931.9(±12.09)	-11.4(±0.63)
10	1404(±10.6)	-3.56(±1.08)
11	709.6(±7.35)	-11.1(±0.6)
12	970.2(±0.46)	-12.4(±0.29)
Drug	708.4(±8.2)	-15.4(±3.1)

The nanoparticles showed a significant change in particle size compared to the untreated drug with the exception of formulation 11 which did not significantly differ from the drug.

As for statistical analysis of the zeta potential, it was found that the drug has a significantly different zeta potential compared to all formulations.

*Saturated Solubility Determination:*

The obtained results for the saturated solubility of the twelve formulations, drug and lyophilized drug are listed in Table 3.

**Table 3:** Saturated Solubility of formulations

**Formulation #**	**Saturated Solubility (**µ**g/mL)**
1	78.55(±2.97)
2	93.28(±12.9)
3	383.95(±4.27)
4	134.59(±3.82)
5	79.98(±1.43)
6	182.78(±3.82)
7	73.16(±1.29)
8	76.14(±2.02)
9	79.25(±6.89)
10	60.35(±1.66)
11	71.18(±1.28)
12	78.75(±0.87)
Drug	43.67(±4.24)
Lyophilized Drug	68.804(±0.85)
Physical mixture for Formulation 3	154.294 (±0.71)

It was clear that the saturated solubility of formulation 3 was significantly higher than that of the drug, lyophilized drug, the rest of the formulations as well as its physical mixture at P < 0.05.

*Dissolution Studies*

**Figure 1:**
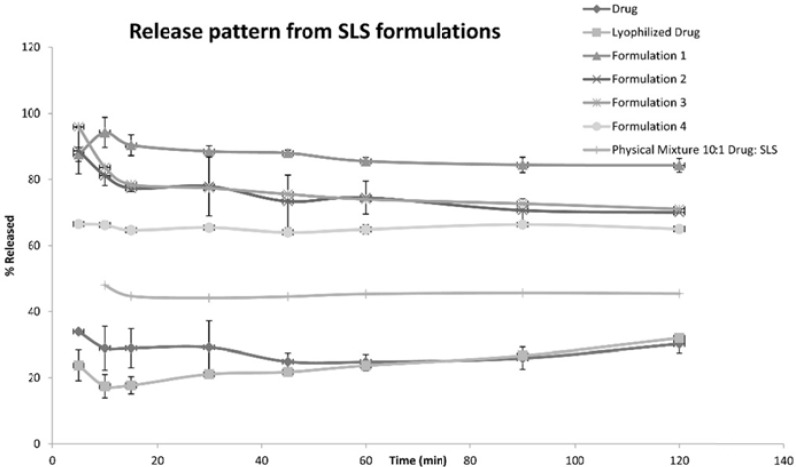
Release Pattern from SLS Formulations

**Figure 2:**
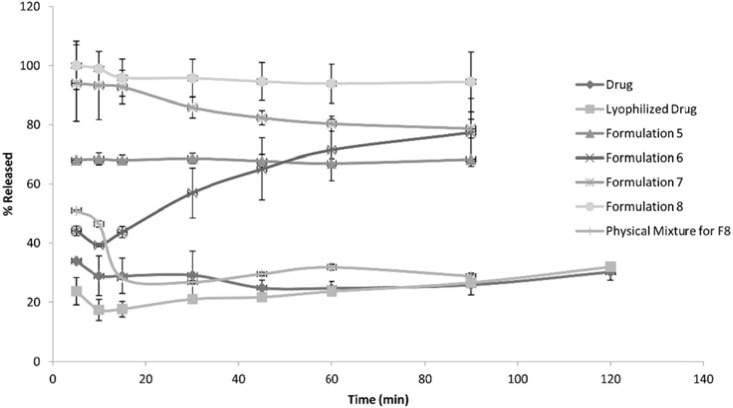
Release Pattern from Tween 80 Formulations

**Figure 3:**
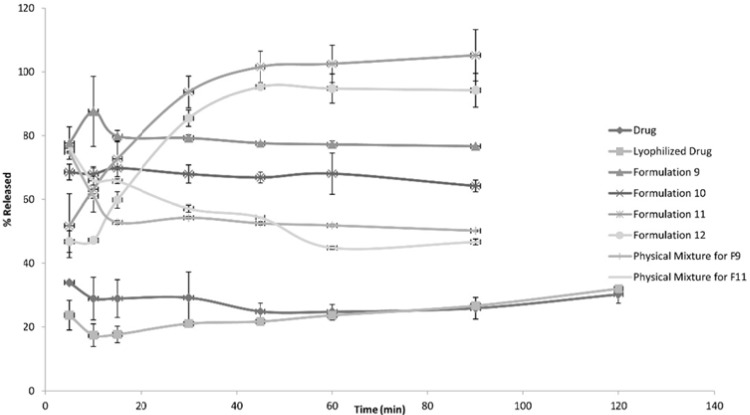
Release Patterns from HPMC and HPC Formulations

The following Figures 1, 2 and 3 show the percentage release from the SLS, Tween 80 and HPMC, HPC formulations, respectively, compared to both the drug, lyophilized drug and the physical mixture of the formulation which showed the highest dissolution rate.

The figures showed a significant increase at p < 0.05, in the percentage of the drug released after 30 min for the nanocrystal formulations (1-12) compared to the drug, the lyophilized drug and the physical mixtures. The lophilization did not significanlty affect the dissolution rate of the drug, where after 120 min the percentage of the drug released did not exceed 32%. The nanocrystal formulations of SLS approached 100% release after ten min. They were also significantly higher compared to that of (drug: SLS 10:1) physical mixture. Tween 80 formulations showed an increase in the percentage released as the amount of added Tween 80 increased in the formulation. As for HPMC and HPC formulations, it was clear that both were superior to the drug and its lyophilized form, yet HPC non significantly improved the dissolution rate compared to HPMC after 30 min from the beginning of the experiment. The formulations also showed an increase in the percentage released compared to the physical mixtures.

Table 4 shows the results for the %D.E. for the plain drug, lyophilized drug, physical mixtures and the twelve formulations. The twelve prepared formulations possessed higher values compared to the plain, lyophilized drugs as well as the the physical mixtures.

**Table 4:** Dissolution Efficiency of nanocrystal formulations compared to the plain drug.

**Formulation #**	**% D.E.**
1	33.791
2	29.881
3	30.399
4	24.567
5	25.647
6	18.246
7	33.727
8	36.358
9	30.411
10	29.106
11	25.669
12	25.125
Drug	11.095
Lyophilized Drug	7.418
Physical mixture for Formulation 3	15.073
Physical mixture for formulation 8	12.183
Physical mixture for formulation 9	21.655
Physical mixture for formulation 11	23.657

*X-Ray Diffraction Analysis*

**Figure 4:**
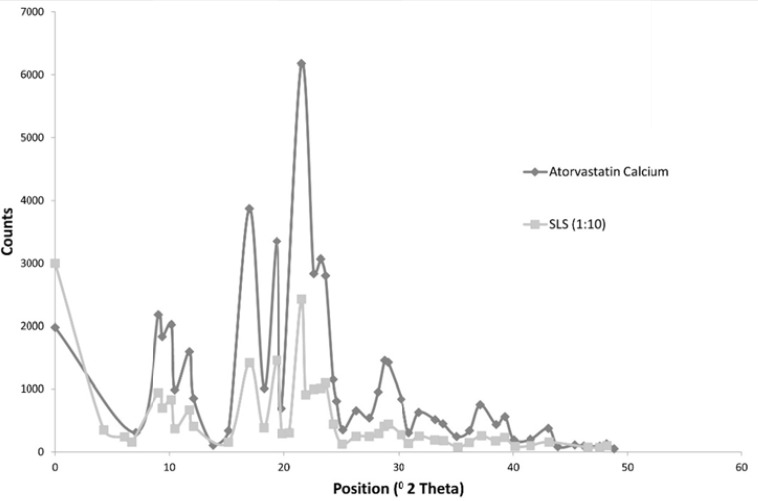
X ray Diffraction Picture of both the Drug and Formulation 3

The following Figure 4 shows the X ray diffraction picture of both the drug and formulation 3 which showed an acceptable release pattern and the highest saturation solubility.

The Figure shows that both the drug and Formulation 3 exhibited diffraction peaks characteristic for crystalline Atorvastatin Calcium at the 2θ degree values of 9.37, 11.76, 12.10 and 16.96° [25]. Some additional peaks were observed at 2θ of 6.0169 and 21.9 for formulation 3. Generally, the intensity of peaks was much decreased for formulation 3.

**Figure 5:**
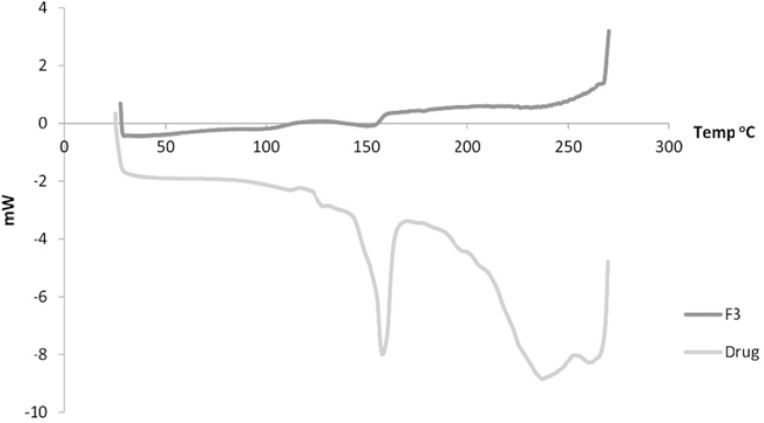
DSC of both the drug and Formulation 3

Figure 5 shows the DSC of both the drug and F3. The drug showed a sharp peak at about 157^ o^C. For Formulation 3, a peak is observed at 153^ o^C.

**Figure 6:**
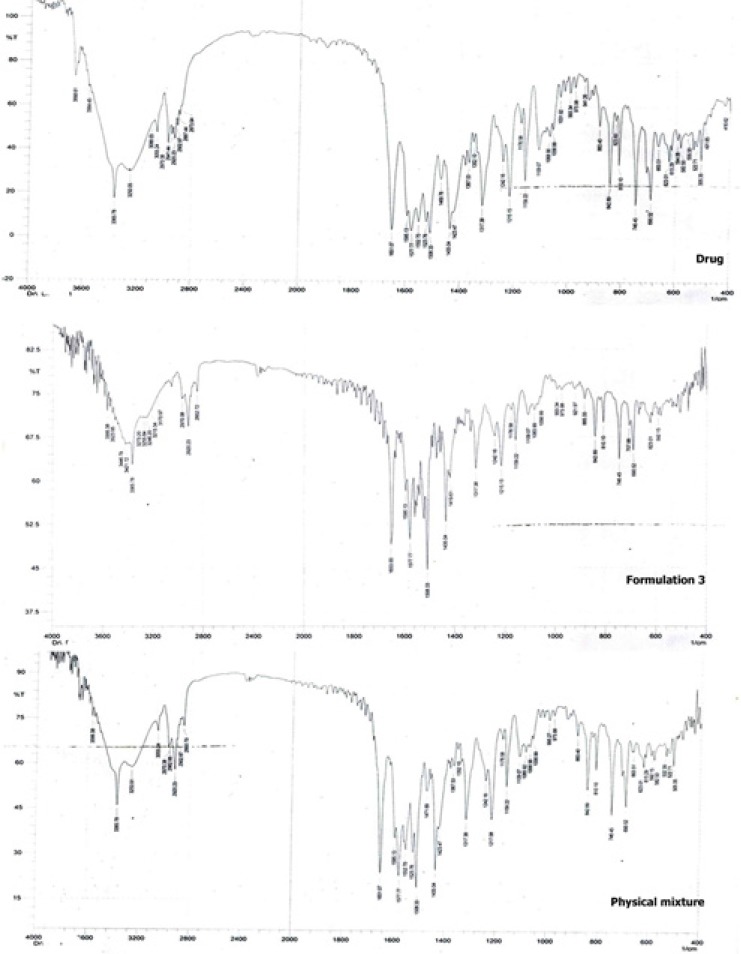
FTIR spectroscopic analysis

Figure 6 shows the FTIR spectroscopic analysis of the drug, formulation 3 and the physical mixture corresponding to formulation 3. The pure drug showed characteristic peaks at 2941 cm^-1^ (C-H - stretching), 1317 cm^-^^1^(C-N - stretching), 3055 cm^-1^ (C-HO-stretching alcoholic group), 1651 cm^-1^ (C = C-bending), 746 cm^-1^ , 690 cm^-1^ (C-F-stretching), 1109 cm^-1^ (O-H-bending) (26). It is clear that the main drug peaks are retained in both the physical mixture as well as the nanocrystals of the drug indicating no possible interaction between the drug and the stabilizer.

*Scanning electron microscopy*

**Figure 7:**
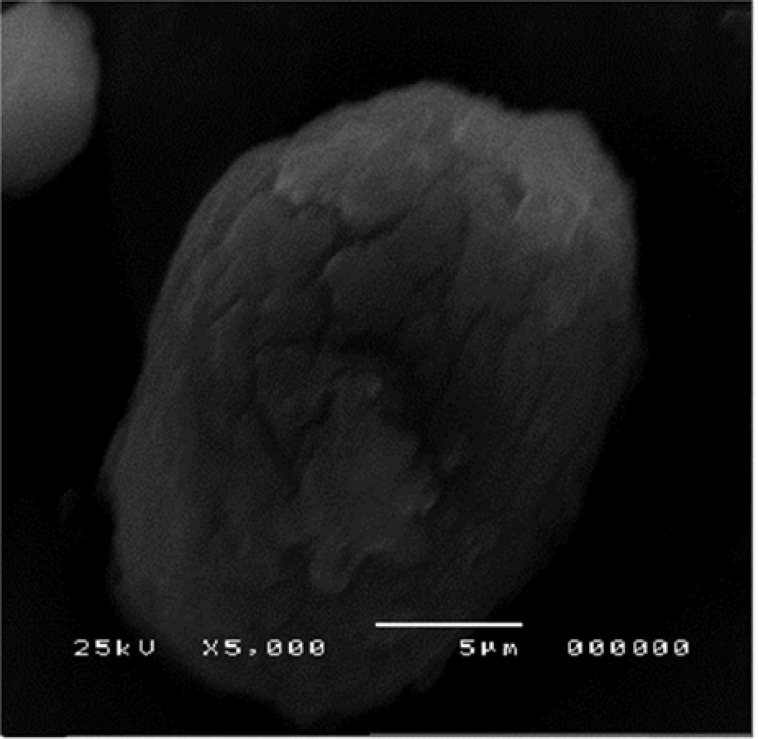
SEM of nanocrystals of Formulation 3

The morphology of the nanocrystals of formulation 3 was observed using high resolution SEM as shown is Figure 7.

**Figure 8:**
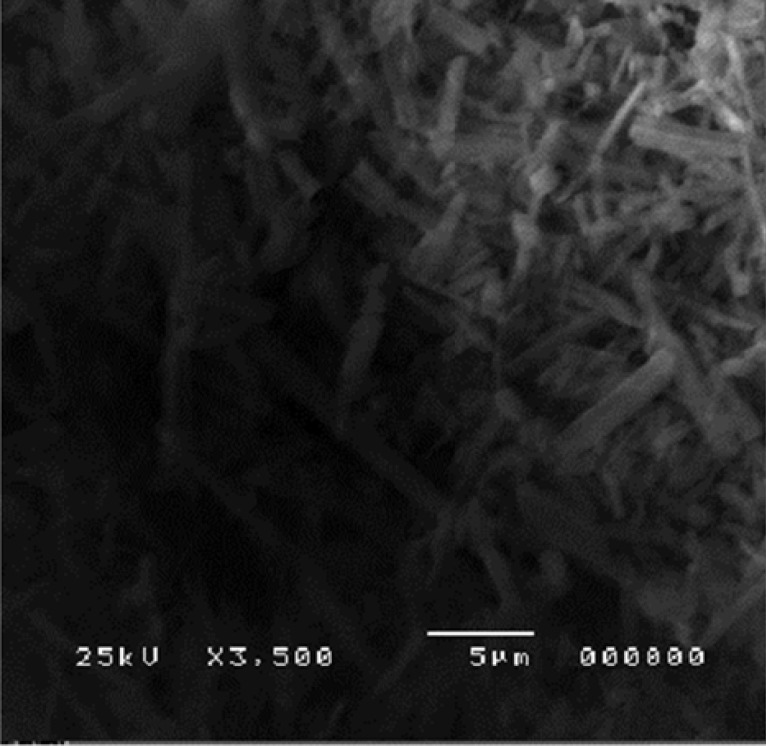
SEM of Atorvastatin Calcium

The agglomerates were composed of spherical nanoparticles. Figure 8 represents the morphology of atorvastatin calcium whose crystals were plate shaped.

*In-vivo study results*

**Figure 9:**
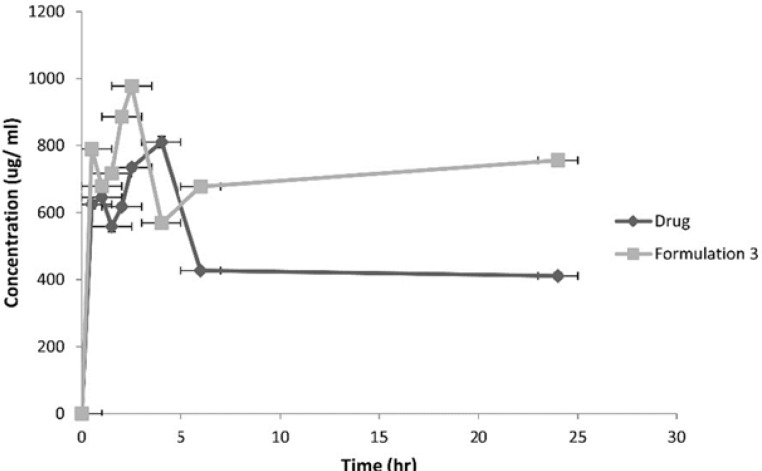
Plasma-Concentration Time Curves of Atorvastatin Calcium and Formulation 3.

Following the administration of the equivalent of 10 mg/1 mL atorvastatin, the pharmacokinetic parameters of both the drug and formulation 3 were calculated. Figure 9 represents the average plasma concentrations of the two groups of rats administering the drug and formulation 3.

**Table 5:** Pharmacokinetic parameters of the drug and Formulation 3 adminstered to rats

**Formulation**	**C**_pmax_ **(**µ**g/mL)**	**T**_max_ **(hour)**	**AUC**_(0-24)_ **(mAU.s)**	**MRT** **(hour)**
Drug	810.15 (± 12.6)	4 (±1.7)	29997.59 (±4.66)	48.16 (±2.105)
Formulation 3	977.51 (±19.4)	2.5 (±0.3)	86429.3 (±5.9)	94.64 (±3.96)

C_Pmax_: maximum plasma concentration; T_max_: Time to maximum plasma concentration; AUC _(0-24)_: Area under plasma concentration-time curve till 24 h; MRT: mean residence time

The AUC _0-24 hr,_ MRT and Cpmax for formulation 3 were significantly higher at p < 0.05 compared to the drug.

The aforementioned results can possibly be explained as follows. A significant change in particle size observed between the different formulations and the drug at p < 0.05 was possibly attributed to the addition of a stabilizer. The surfactants (SLS and Tween 80) and the polymers (HPMC and HPC), were able to keep the size reduced particles–due to homogenization-at a distance sufficient to avoid predominance of the attraction forces that can result in irreversible aggregation. This sufficient distance was a result of one of the following causes. The repulsive forces generated on the particle surface achieved by surfactants that cover particle surface, or due to the steric stabilization generated by the polymer use (10, 20).

The best stabilization can be obtained at the optimal stabilizer concentration, where too little stabilizer resulted in particle agglomeration, and too much stabilizer resulted in Ostwald ripening which is small crystals dissolving and rejoining larger ones to give larger crystals (27). This optimal concentration was achieved with the formulation 3 in the SLS formulations (anionic surfactant) and formulation 8 in the Tween 80 formulations (non-ionic surfactant).

Formulation 3, also, showed optimum zeta potential - 25.7(± 0.42) (10). As well as the highest saturation solubility where there was about a nine- fold increase compared to that of Atorvastatin Calcium. It has got a rapid drug release pattern which was significantly higher than the drug and its lyophilized form.

For the Tween 80 formulations, the increase in Tween 80 concentration was associated by a corresponding particle size reduction. This was probably due to a densely packed, thicker adsorbed layer of Tween 80 (28).

A significant change in particle size with HPMC and HPC was not observed probably due to the relatively high viscosity of the resulting suspension. Thickening of the milled product is a major concern. There is always a possibility that the flow through the recirculation loop of the homogenizer can cease due to excessive yield stress. Also, the high suspension viscosity can stop the milling operation (29). This in turn resulted in the relatively large particle size of formulations 9, 10, 11 and 12. Also these formulations were not suitable for further investigation due to their low zeta potential (< 20 mV) which reflected unstable nano suspensions highly susceptible to undergo aggregation (30).

HPMC and HPC act by surrounding fine drug crystals, thus they hinder their re-crystallization from solution by reducing the surface area for crystallization on the drug particles, and hence reduce crystal growth. This, however, hindered dissolution by forming a barrier against penetration of water molecules. This resulted in their relatively slow dissolution rate compared to formulations containing SLS and Tween (31).

Thus formulation 3 was found to be most suitable to carry out further investigation as X-ray diffraction analysis, SEM and the *in-vivo* study.

X-ray diffraction analysis showed retention of the crystalline state of the drug after high pressure homogenization, since the diffraction pattern was preserved. The additional peaks observed for the formulation 3 are characteristic peaks for SLS which is adsorbed on the surface of crystals (32). Also the observed decrease in the intensity of the peaks for formulation 3 was due to the decrease of particle size compared to drug (33). 

In the DSC, the observed reduction in the endothermic peak of formulation 3 indicates possibly transformation to a more amorphous form of the drug which exhibits polymorphism (25). 

SEM showed the surface morphology of Formulation 3 as spherical particles compared to the drug.

As for the *in-vivo* study, it revealed an improvement in bioavailability of atorvastatin calcium due to nanosizing. It was thought that nanosuspensions had general adhesiveness to the intestinal wall due to their large surface area. The adhesiveness of nanoparticles in intestine would increase the passive absorption (34). Also, this adhesiveness resulted in increasing the residence time in the gastrointestinal tract leading to improved bioavailability (35). The rapid attainment of peak plasma concentrations was due to the burst release effect brought about by the use of SLS for stabilization of nanocrystals (36).

It was observed that after oral dosing of the drug and the formulation 3, their individual kinetic curve exhibited double peaks. The presence of enterohepatic cycling is not an acceptable explanation as atorvastatin does not exhibit such phenomenon (37). Thus double peaks can be due to the existence of two absorption sites in the gut interrupted by a region of poor absorption (38).
